# Supplementary material for: EnzML: multi-label prediction of enzyme classes using InterPro signatures
Source: BMC Bioinformatics. 2012 Apr 25;13:61. doi: 10.1186/1471-2105-13-61 (PMC3483700; doi:10.1186/1471-2105-13-61)
Supplement: Addtional file 5 — The Java code to format the data files, evaluate and predict. The file enzml_java_code.tar.gz contains the Java code used to format database data to ARFF and XML formats, to execute cross and train-test (jackknife) evaluations and to record evaluation results to database. More information is included in the readme.txt file and the Javadoc files. The code can be used with a MySQL database. To use a different database software, other JDBC drivers might be required. [file 1471-2105-13-61-S5.gz › java_code/enzml2011/doc/uk/ac/ed/inf/enzml/Path.html]

Path


---


|  |  |  |
| --- | --- | --- |
| **Overview** \| **Package** \| **Class** \| **Use** \| **Tree** \| **Deprecated** \| **Index** \| **Help** | |  |
| PREV CLASS   NEXT CLASS | **FRAMES**    **NO FRAMES**     **All Classes** |  |
| SUMMARY: NESTED \| FIELD \| CONSTR \| METHOD | DETAIL: FIELD \| CONSTR \| METHOD |  |


---


## uk.ac.ed.inf.enzml Class Path

```
java.lang.Object
  uk.ac.ed.inf.enzml.Path
```

---

``` public class Path extends java.lang.Object ```

Contains file paths (database connection files, arff properties files, mulan
xml files etc.)

**Version:**
:   14 Jun 2010

**Author:**
:   Luna De Ferrari luna.deferrari-at-ed.ac.uk

---

| **Field Summary** | |
| --- | --- |
| `static java.lang.String` | `ARFF_FILES_FILE_DIR` |
| `static java.lang.String` | `ARFF_PROPERTIES_FILE_DIR` |
| `static java.lang.String` | `ARFF_PROPS_ARCHAEA` |
| `static java.lang.String` | `ARFF_PROPS_BACTERIA` |
| `static java.lang.String` | `ARFF_PROPS_EUKARIA` |
| `static java.lang.String` | `ARFF_PROPS_FUNGI` |
| `static java.lang.String` | `ARFF_PROPS_HUMAN` |
| `static java.lang.String` | `ARFF_PROPS_INVERTEBRATES` |
| `static java.lang.String` | `ARFF_PROPS_NOT_HUMAN`             all swissprot-kegg agree records, except human |
| `static java.lang.String` | `ARFF_PROPS_PATH` |
| `static java.lang.String` | `ARFF_PROPS_PLANTS` |
| `static java.lang.String` | `ARFF_PROPS_RANDOM_100` |
| `static java.lang.String` | `ARFF_PROPS_RANDOM_ARCHAEA` |
| `static java.lang.String` | `ARFF_PROPS_RANDOM_BACTERIA` |
| `static java.lang.String` | `ARFF_PROPS_RANDOM_EUKARIA` |
| `static java.lang.String` | `ARFF_PROPS_RANDOM_FUNGI` |
| `static java.lang.String` | `ARFF_PROPS_RANDOM_INVERTEBRATES` |
| `static java.lang.String` | `ARFF_PROPS_RANDOM_PLANTS` |
| `static java.lang.String` | `ARFF_PROPS_RANDOM_VERTEBRATES` |
| `static java.lang.String` | `ARFF_PROPS_SWISSKEGG` |
| `static java.lang.String` | `ARFF_PROPS_TREMBL_KEGG` |
| `static java.lang.String` | `ARFF_PROPS_VERTEBRATES` |
| `static java.lang.String` | `BASE_PATH` |
| `static java.lang.String` | `MACHINE_LEARNING_LOCAL_DB_PROPS` |
| `static java.lang.String` | `MACHINE_LEARNING_PROPS` |
| `static java.lang.String` | `MACHINE_LEARNING_REMOTE_DB_PROPS` |
| `static java.lang.String` | `ML_PROPERTIES_FILE_DIR` |
| `static java.lang.String` | `MODEL_FOLDER` |
| `static java.lang.String` | `PREDICTIONS_FOLDER` |
| `static java.lang.String` | `PROJECT_NAME` |
| `static java.lang.String` | `PROPERTIES_FILE_DIR` |
| `static java.lang.String` | `RESULTS_DIRECTORY` |


**Constructor Summary**

`Path()`


**Method Summary**

**Methods inherited from class java.lang.Object**

`equals, getClass, hashCode, notify, notifyAll, toString, wait, wait, wait`

**Field Detail**

### BASE\_PATH

```
public static final java.lang.String BASE_PATH
```

**See Also:**: Constant Field Values

---


### MODEL\_FOLDER

```
public static final java.lang.String MODEL_FOLDER
```

**See Also:**: Constant Field Values

---


### PREDICTIONS\_FOLDER

```
public static final java.lang.String PREDICTIONS_FOLDER
```

**See Also:**: Constant Field Values

---


### PROJECT\_NAME

```
public static final java.lang.String PROJECT_NAME
```

**See Also:**: Constant Field Values

---


### PROPERTIES\_FILE\_DIR

```
public static final java.lang.String PROPERTIES_FILE_DIR
```

**See Also:**: Constant Field Values

---


### ARFF\_FILES\_FILE\_DIR

```
public static final java.lang.String ARFF_FILES_FILE_DIR
```

**See Also:**: Constant Field Values

---


### ARFF\_PROPERTIES\_FILE\_DIR

```
public static final java.lang.String ARFF_PROPERTIES_FILE_DIR
```

**See Also:**: Constant Field Values

---


### ARFF\_PROPS\_PATH

```
public static final java.lang.String ARFF_PROPS_PATH
```

**See Also:**: Constant Field Values

---


### ML\_PROPERTIES\_FILE\_DIR

```
public static final java.lang.String ML_PROPERTIES_FILE_DIR
```

**See Also:**: Constant Field Values

---


### RESULTS\_DIRECTORY

```
public static final java.lang.String RESULTS_DIRECTORY
```

**See Also:**: Constant Field Values

---


### ARFF\_PROPS\_ARCHAEA

```
public static final java.lang.String ARFF_PROPS_ARCHAEA
```

**See Also:**: Constant Field Values

---


### ARFF\_PROPS\_BACTERIA

```
public static final java.lang.String ARFF_PROPS_BACTERIA
```

**See Also:**: Constant Field Values

---


### ARFF\_PROPS\_EUKARIA

```
public static final java.lang.String ARFF_PROPS_EUKARIA
```

**See Also:**: Constant Field Values

---


### ARFF\_PROPS\_FUNGI

```
public static final java.lang.String ARFF_PROPS_FUNGI
```

**See Also:**: Constant Field Values

---


### ARFF\_PROPS\_HUMAN

```
public static final java.lang.String ARFF_PROPS_HUMAN
```

**See Also:**: Constant Field Values

---


### ARFF\_PROPS\_NOT\_HUMAN

```
public static final java.lang.String ARFF_PROPS_NOT_HUMAN
```

:   all swissprot-kegg agree records, except human

    **See Also:**: Constant Field Values

---


### ARFF\_PROPS\_INVERTEBRATES

```
public static final java.lang.String ARFF_PROPS_INVERTEBRATES
```

**See Also:**: Constant Field Values

---


### ARFF\_PROPS\_PLANTS

```
public static final java.lang.String ARFF_PROPS_PLANTS
```

**See Also:**: Constant Field Values

---


### ARFF\_PROPS\_SWISSKEGG

```
public static final java.lang.String ARFF_PROPS_SWISSKEGG
```

**See Also:**: Constant Field Values

---


### ARFF\_PROPS\_TREMBL\_KEGG

```
public static final java.lang.String ARFF_PROPS_TREMBL_KEGG
```

**See Also:**: Constant Field Values

---


### ARFF\_PROPS\_RANDOM\_100

```
public static final java.lang.String ARFF_PROPS_RANDOM_100
```

**See Also:**: Constant Field Values

---


### ARFF\_PROPS\_RANDOM\_ARCHAEA

```
public static final java.lang.String ARFF_PROPS_RANDOM_ARCHAEA
```

**See Also:**: Constant Field Values

---


### ARFF\_PROPS\_RANDOM\_BACTERIA

```
public static final java.lang.String ARFF_PROPS_RANDOM_BACTERIA
```

**See Also:**: Constant Field Values

---


### ARFF\_PROPS\_RANDOM\_EUKARIA

```
public static final java.lang.String ARFF_PROPS_RANDOM_EUKARIA
```

**See Also:**: Constant Field Values

---


### ARFF\_PROPS\_RANDOM\_FUNGI

```
public static final java.lang.String ARFF_PROPS_RANDOM_FUNGI
```

**See Also:**: Constant Field Values

---


### ARFF\_PROPS\_RANDOM\_INVERTEBRATES

```
public static final java.lang.String ARFF_PROPS_RANDOM_INVERTEBRATES
```

**See Also:**: Constant Field Values

---


### ARFF\_PROPS\_RANDOM\_PLANTS

```
public static final java.lang.String ARFF_PROPS_RANDOM_PLANTS
```

**See Also:**: Constant Field Values

---


### ARFF\_PROPS\_RANDOM\_VERTEBRATES

```
public static final java.lang.String ARFF_PROPS_RANDOM_VERTEBRATES
```

**See Also:**: Constant Field Values

---


### ARFF\_PROPS\_VERTEBRATES

```
public static final java.lang.String ARFF_PROPS_VERTEBRATES
```

**See Also:**: Constant Field Values

---


### MACHINE\_LEARNING\_LOCAL\_DB\_PROPS

```
public static final java.lang.String MACHINE_LEARNING_LOCAL_DB_PROPS
```

**See Also:**: Constant Field Values

---


### MACHINE\_LEARNING\_REMOTE\_DB\_PROPS

```
public static final java.lang.String MACHINE_LEARNING_REMOTE_DB_PROPS
```

**See Also:**: Constant Field Values

---


### MACHINE\_LEARNING\_PROPS

```
public static final java.lang.String MACHINE_LEARNING_PROPS
```

**See Also:**: Constant Field Values


**Constructor Detail**

### Path

```
public Path()
```


---


|  |  |  |
| --- | --- | --- |
| **Overview** \| **Package** \| **Class** \| **Use** \| **Tree** \| **Deprecated** \| **Index** \| **Help** | |  |
| PREV CLASS   NEXT CLASS | **FRAMES**    **NO FRAMES**     **All Classes** |  |
| SUMMARY: NESTED \| FIELD \| CONSTR \| METHOD | DETAIL: FIELD \| CONSTR \| METHOD |  |


---
